# Pneumococcal carriage in unvaccinated children at the time of vaccine implementation into the national immunization program in Poland

**DOI:** 10.1038/s41598-022-09488-z

**Published:** 2022-04-07

**Authors:** Izabela Wróbel-Pawelczyk, Patrycja Ronkiewicz, Monika Wanke-Rytt, Dominika Rykowska, Aneta Górska-Kot, Katarzyna Włodkowska, Agnieszka Topczewska-Cabanek, Teresa Jackowska, Joanna Chruszcz, Walentyna Marchut, Agnieszka Mastalerz-Migas, Krzysztof Korzeniewski, GIL Study Team, Karolina Gastoł, Marta Gromek, Katarzyna Jankowska-Sasin, Katarzyna Karpierz, Magdalena Okarska-Napierała, Dagmara Pokorna-Kałwak, Agata Polit, Julia Robakiewicz, Maciej Rygalski, Anna Siwonia, Martyna Strzałka, Leszek Szenborn, Martyna Szwejkowska, Zofia Szymańska-Toczek, Izabela Zaleska, Katarzyna Żołnierowicz, Anna Skoczyńska, Krzysztof Trzciński

**Affiliations:** 1 Department of Epidemiology and Clinical Microbiology, National Medicines Institute, Chełmska 30/34, 00-725 Warsaw, Poland; 2 Department of Pediatrics with Clinical Assessment Unit, Medical University of Warsaw, Warsaw, Poland; 3 Niekłańska Children’s Hospital, Pediatrics, Warsaw, Poland; 4 Medical Centre MEDICERS Zacisze, Warsaw, Poland; 5 Department of Family Medicine, Medical University of Warsaw, Warsaw, Poland; 6 Department of Pediatrics, The Centre of Postgraduate Medical Education, Warsaw, Poland; 7 Department of Pediatric Infectious Diseases, Wroclaw Medical University, Wrocław, Poland; 8 Białołęka Medical Centre, Warsaw, Poland; 9 Department of Family Medicine, Wroclaw Medical University, Wrocław, Poland; 10 Department of Epidemiology and Tropical Medicine, Military Institute of Medicine, Warsaw, Poland; 11 Department of Tropical Medicine and Epidemiology, Institute Maritime and Tropical Medicine, Medical University of Gdansk, Gdańsk, Poland; 12 Department of Pediatric Immunology and Infectious Diseases, Wilhelmina Children’s Hospital, University Medical Center Utrecht, Utrecht, The Netherlands; 13 Department of Pediatrics, Bielański Hospital, Warsaw, Poland; 14 Department of Pediatrics with Pediatric Neurosurgery Unit, Medical University of Warsaw, Warsaw, Poland; 15 Medical Centre AD-MED, Wrocław, Poland

**Keywords:** Bacteria, Infectious-disease epidemiology

## Abstract

We investigated pneumococcal carriage among unvaccinated children under five years of age at a time when the conjugate polysaccharide vaccine (PCV) was introduced in Poland into the national immunization program (NIP). Paired nasopharyngeal swab (NPS) and saliva samples collected between 2016 and 2020 from n = 394 children were tested with conventional culture and using qPCR. The carriage rate detected by culture was 25.4% (97 of 394), by qPCR 39.1% (155 of 394), and 40.1% (158 of 394) overall. The risk of carriage was significantly elevated among day care center attendees, and during autumn/winter months. Among isolates cultured, the most common serotypes were: 23A, 6B, 15BC, 10A, 11A. The coverage of PCV10 and PCV13 was 23.2% (23 of 99) and 26.3% (26 of 99), respectively. Application of qPCR lead to detection of 168 serotype carriage events, with serogroups 15, 6, 9 and serotype 23A most commonly detected. Although the highest number of carriers was identified by testing NPS with qPCR, saliva significantly contributed to the overall number of detected carriers. Co-carriage of multiple serotypes was detected in 25.3% (40 of 158) of carriers. The results of this study represent a baseline for the future surveillance of effects of pneumococcal vaccines in NIP in Poland.

## Introduction

*Streptococcus pneumoniae* is the common cause of invasive bacterial disease^1,2^. Incidence of invasive pneumococcal disease (IPD) is highest among infants, toddlers and in older adults^1^. Despite available vaccines, in 2015 pneumococcus was responsible globally for approximately 300,000 deaths of children under 5 years of age^2^. IPD is manifested by meningitis, sepsis and/or bacteremic pneumonia. *S. pneumoniae* also causes milder infections manifested across all ages as sinusitis or non-bacteremic pneumonia, and in children as acute otitis media. The primary virulence factor of pneumococci is the polysaccharide capsule^3^ and currently available pneumococcal vaccines are all based on capsular polysaccharides as the antigen. While there have been over 100 capsular types (serotypes) described^4,5^, marketed vaccines target only a subset of ten to twenty-three serotypes (24 serotypes in total). Pneumococcal conjugate vaccines (PCV) recommended for children^2^, target ten (PCV10) and thirteen (PCV13) serotypes common in paediatric disease prior to PCVs introduction. PCV10 and PCV13 have been commercially available in Poland since 2009 and 2010, respectively. In 2017, PCV10 was introduced into the National Immunization Program (NIP) for all children born after the 31st of December 2016, and PCV10 was chosen as the refunded vaccine in all consecutive annual NIP tenders. Children are vaccinated at 2, 4, and 13–15 months of life (2 + 1 schedule). However, it is estimated that a quarter to a third of infants in Poland is vaccinated with PCV13 outside the NIP^6^.

Three years after PCV10 implementation into NIP, there was a significant decrease of IPD cases caused in Poland by PCV10 vaccine serotypes (VTs) in children under 2 years of age (57% in years 2014–2016 vs. 31% in years 2017–2019)^7^. There was also a significant decline from 35 to 28% in PCV10-VTs IPDs in persons ≥ 65 years old. Similar herd effects were earlier observed in other countries^8–10^ and are attributed to PCVs preventing VT strains carriage acquisition in vaccinees^11,12^. Since children are the main reservoir and the main transmitters of pneumococci, infants’ vaccination with PCVs may have an impact on carriage and disease in unvaccinated individuals in the same population^2,3,13^. Consequently, effects of PCVs can be monitored not only via the surveillance of disease but also of carriage^14^.

Since 1997 the National Reference Centre for Bacterial Meningitis (NRCBM) collects isolates causing IPD from the whole Poland and also conducts molecular diagnostics of IPD cases. Although the surveillance of IPD in the country is well established, the data on *S. pneumoniae* and pneumococcal serotypes carriage is limited. Over the past twenty years there have been only two studies published on pneumococcal carriage conducted in Poland^15–18^. To fill the gap, we investigated the pneumococcal carriage in unvaccinated children under five years of age. Our goal was to map the carriage of *S. pneumoniae* serotypes in the paediatric population before and right after the nationwide immunization of infants with PCV10, when herd effects might not be substantial yet. With this, we were aiming to establish a baseline for future surveillance studies.

The gold standard for pneumococcal carriage detection is the isolation of viable *S. pneumoniae* from a culture of a deep trans-nasal nasopharyngeal swab (NPS)^19,20^. There is evidence that other samples, oral fluids in particular, may serve as a substitute for NPS^21,22^. Compared to NPS, saliva is much easier to collect, can be even self-collected, and, except for the youngest children, it does not require a designated collection kit. It has been also reported that molecular methods are more sensitive in detecting *S. pneumoniae* and pneumococcal serotype carriage^23–25^. Hence, our second goal was to compare results of carriage detection by testing saliva versus NPS and using molecular methods versus conventional culture in order to develop a procedure tailored to our future studies^26–28^. To our knowledge, there are no published studies comparing saliva testing with the gold standard in children under five years of age.

## Materials and methods

### Study design

The study was performed between August 2016 and March 2020 among children between the ages of 12–59 months. The children were not vaccinated with any pneumococcal vaccine and attended a ‘non-sick-visit’ in hospital outpatients’ clinics or community health-care centers in the cities of Warsaw and Wroclaw. All parents (or the child’s legal guardians) were asked if the family would be interested in participating in the study. If they responded positively, they were informed about the study goals and procedures and asked to give written informed consent for the child’s participation. Next, parents were asked to fill-in the questionnaire and provide information on the child’s age, sex, environment (number of siblings, day-care attendance, and the presence of a smoker in child’s household), and clinical information (pneumococcal vaccination, reason for doctor’s office visit, occurrence of chronic diseases, symptoms of lower respiratory tract infections, antibiotic therapy in the past three months). The questionnaire was reviewed on site by the study personnel to be sure to exclude children who have been vaccinated with any pneumococcal vaccine, were treated within last 4 weeks with any antibiotic, or had any immunodeficiency or symptoms of lower respiratory tract infections. Finally, first a saliva sample and then nasopharyngeal swab was collected from each child by the study’s medical personnel.

### Ethical statement

The study was approved by the Bioethics Committee of the Military Institute of Medicine in Warsaw (Resolution No. 49/WIM/2016) and was conducted in accordance with the World Health Medical Association 1964 Declaration of Helsinki and the EU rules of Good Clinical Practice.

### Collection of samples

Saliva samples were collected using the Oracol+^®^ kit (Malvern Medical Developments Ltd., UK) as previously described^26^. NPS’s were collected using ESwab™ 482C (COPAN Diagnostics, Brescia, Italy) and immediately placed in 1 ml Amies transport medium (COPAN Diagnostics). Within 32 h samples were delivered to the NRCBM and either immediately cultured or supplemented on arrival with 10% glycerol and stored at − 70 °C.

### Culturing samples for *S. pneumoniae*

NPS samples and Oracol+^®^ saliva samples were plated on Columbia Agar (Becton Dickinson, USA) 7% Sheep blood (Graso Biotech, Poland) with 5 µg/ml gentamicin (Sigma-Aldrich, USA) (GENT-agar) and incubated for 18–24 h in 35 °C, 5% CO_2_ as previously described^21,29^. Once pneumococcus-like colonies were re-plated, all remaining colony growth was harvested into 2.1 ml Brain Heart Infusion (Graso Biotech, Poland) with 10% glycerol^21^. These harvests represented samples culture-enriched (CE) for *S. pneumoniae*^29^. Re-plated isolates were identified as *S. pneumoniae* based on susceptibility to optochin (BioMerièux, France) and bile solubility (Becton Dickinson, USA)^30^.

### Molecular detection of *S. pneumoniae*

The DNA was extracted from 200 µl of culture-enriched NPS’s and culture-enriched saliva samples using the SaMag-12™ System (Sacace Biotechnologies, Italy). The DNA was eluted into 100 µl sample volume and stored in 2–8 °C. Pneumococcal DNA was detected with qPCR by testing on a 7500 Real Time PCR System (Life Technologies, USA) a 0.625 μl of eluted sample in 25 μl reaction volume, using TaqMan Universal MasterMix (Life Technologies, USA), and primers and probes targeting sequences within *piaB* and *lytA* genes^29,31^. A sample was considered positive for pneumococcus when both signals were < 40 C_*T*_^21,23,32^.

### Serotyping of *S. pneumoniae* isolates

All cultured pneumococci were first serotyped using the ImmuLex™ Pneumotest Kit (SSI Diagnostica, Denmark), supplemented with a PCR and gene sequencing as previously described^33–35^. Serotypes not identified by the above methods were subjected to the Neufeld Quellung test^36^.

### Molecular detection of *S. pneumoniae* serotypes

DNA extracted from culture-enriched NPS’s and saliva was tested with qPCR for the presence of serotype-specific sequences by testing 1.25 μl of sample in 12.5 μl reaction volume in duplicate, as described before^37^, using TaqMan Universal MasterMix (Life Technologies, USA), primers and probes for serotype 1, 3, 4, 5, 6A/B/C/D, 7A/F, 8, 9A/L/N/V, 10A/B, 12A/B/F, 14, 15A/BC/F, 19A, 20, 21, 22A/F, 23A, 23B, 23F, 33A/F, 35B/C, and 38 published by Azzari et al*.*^38^, for serotype 16F published by Azzari et al*.*^39^, for serotypes 11A/D, 18A/B/C/F, and 19F published by Pimenta et al*.*^40^, and for serotype 34 published by Sakai et al*.*^41^. A sample was considered positive for pneumococcus when the signal detected in serotype-specific qPCR was < 40 C_*T*_. All culture-enriched samples classified with molecular methods as positive and two hundred randomly selected culture-enriched samples classified as negative for *S. pneumoniae* (100 NPS’s and saliva samples each) have been tested with qPCR for serotype detection. Assays generating a positive signal in samples classified as negative for pneumococcus were considered as unreliable. In line with previous reports^27,37^, qPCRs targeting serotypes 4 and 5 showed a lack of specificity when applied to both NPS’s and saliva samples. The results of these two assays were excluded from analysis.

### Statistics

The Fisher Exact, and McNemar's tests were used to determine statistical significance (*p* < 0.05). The Chi-square test was used only once when sample size was too large for the Fisher test. In each place where the *p* value was given and it was calculated not with the Fisher test, the name of the statistical test used was added. Data on serotype prevalence detected using different methods was correlated using Spearman’s correlation test for nonparametric data. Test parameters (predictive values, sensitivity, and specificity) were calculated using online calculator (https://www.medcalc.org/calc/diagnostic_test.php).

## Results

Altogether 405 children have been enrolled in the study. Of these, nine children were excluded from further analysis for either being too young (n = 4), too old (n = 4), or being diagnosed with a lower respiratory tract infection on enrolment day (n = 1). Two children were enrolled twice in the study and results of the first sampling were the only included. We report results from 394 children from whom paired NPS and saliva were collected at the same point of time.

### Study population

The frequency of inclusions declined over the study years (Table 1) with half of the children enrolled by the 14th month (September 2017) of the 44-month-long project. The number of 12–23-month-old children (n = 147 of 394) was significantly higher compared with any other age group (*p* < 0.001), and the number of 24–35 months old (n = 102) was significantly higher compared to 36–47 month and 48–59 month-olds (n = 71 and n = 74, respectively, *p* < 0.01).

**Table 1:** Effect of study period, child’s age, sex and environmental factors on *S. pneumoniae* carriage detected using culture and qPCR-based method.

Attribute	Number of individuals	Carriers detected with:				
		Conventional culture		qPCR		Overall
		NPS n = 94	Saliva n = 6	NPS n = 121	Saliva n = 93	n = 158
**Study period**						
Aug 2016–Jul 2017	160	40 (25%)	6 (4%)	60 (38%)	46 (29%)	79 (49%)
Aug 2017–Jul 2018	106	23 (22%)	0	27 (25%)	19 (18%)	31 (29%)
Aug 2018–Jul 2019	97	24 (25%)	0	25 (26%)	23 (28%)	37 (38%)
Aug 2019–Mar 2020	31	7 (23%)	0	9 (29%)	5 (16%)	11 (35%)
**Seasons**						
Spring–Summer	153	28 (18%)	1 (1%)	36 (24%)	25 (16%)	46 (30%)
Autumn–Winter	241	66 (27%)* RR 1.50 (1.01–2.22)	5 (2%) RR 3.17 (0.37–26.9)	85 (35%)** RR 1.50 (1.07–2.09)	68 (28%)** RR 1.73 (1.14–2.61)	112 (46%)*** RR 1.55 (1.17–2.04)
**Child’s age in months**						
12–23	147	42 (29%)	2 (1%)	52 (35%)	34 (23%)	62 (42%)
24–35	102	23 (23%)	0	28 (27%)	20 (20%)	36 (35%)
36–47	71	14 (20%)	2 (3%)	20 (28%)	19 (27%)	30 (42%)
48–59	74	15 (20%)	2 (3%)	21 (28%)	20 (27%)	30 (41%)
**Child’s sex**						
Female	169	43 (25%)	2 (1%)	51 (30%)	41 (24%)	62 (37%)
Male	225	51 (23%) RR 0.89 (0.63–1.27)	4 (2%) RR 1.50 (0.28–8.11)	70 (31%) RR 1.03 (0.76–1.39)	52 (23%) RR 0.95 (0.67–1.36)	96 (43%) RR 1.16 (0.91–1.49)
**Day-care centre attendance**						
No	187	28 (15%)	0	39 (21%)	22 (12%)	49 (26%)
Yes	207	66 (32%)**** RR 2.13 (1.43–3.16)	6 (3%)*	82 (40%)**** RR 1.90 (1.37–2.63)	71 (34%)**** RR 2.92 (1.89–4.51)	109 (53%)**** RR 2.01 (1.53–2.64)
**Smoker in a household**						
No	254	69 (27%)	6 (2%)	87 (34%)	66 (26%)	110 (43%)
Yes	139	25 (18%)* RR 0.66 (0.44–1.00)	0	34 (24%) RR 0.71 (0.51–1.00)	27 (19%) RR 0.75 (0.50–1.11)	48 (35%) RR 0.80 (0.61–1.04)
Unknown	1	0	0	0	0	0
**Siblings**						
No	153	28 (18%)	2 (1%)	39 (25%)	37 (24%)	53 (35%)
Yes	240	66 (28%)* RR 1.50 (1.01–2.23)	4 (2%)	82 (34%) RR 1.34 (0.97–1.85)	56 (23%) RR 0.96 (0.67–1.39)	105 (44%) RR 1.26 (0.97–1.64)
Unknown	1	0	0	0	0	0

*p*—* < 0.05, ** < 0.01, *** < 0.001, and **** < 0.0001.

### Carriage of *S. pneumoniae*

**Figure 1:**
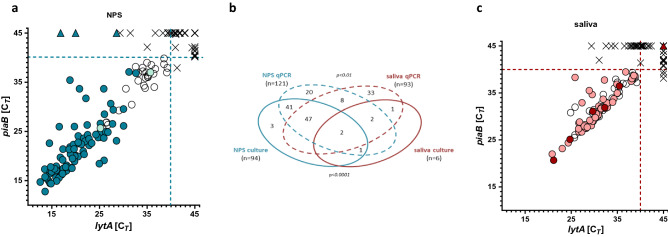
Detection of S*treptococcus pneumoniae* with molecular methods versus isolation of viablei pneumococci from nasopharyngeal (NPS) and saliva samples collected from 394 children. Left and right panels represent scatter plots of the *lytA* and *piaB* qPCR cycle threshold (C_*T*_) values from (**a**) NPS and (**c**) saliva samples. Venn diagram in panel (**b**) depicts the numbers of NPS and saliva samples positive for pneumococci based on recovery of *S. pneumoniae* from a culture (solid-line ovals) or when tested with qPCRs (dashed-line ovals). Each symbol in panels (**a**) and (**c**) represents an individual sample. Samples with a C_*T*_ for both *piaB* and *lytA* below 40 C_T_ (marked with dashed lines) were considered positive for pneumococcus with molecular method. Dark-blue dots in panel (**a**), and dark-red dots in panel (**c**) represent NPS (n = 94) and saliva (n = 6) samples, respectively, from which pneumococci have been cultured. Light-blue dots in panel (**a**) (n = 2), and light-red dots in panel (**c**) (n = 52) represent samples positive for *S. pneumoniae* by molecular method, negative by culture yet with pneumococcus isolated from a paired sample (samples from culture-confirmed carriers). Open circles represent NPS (n = 28), and saliva (n = 41) samples classified with molecular method as positive for *S. pneumoniae* from children from whom no *S. pneumoniae* isolate has been cultured. Triangles represent NPS (n = 3) and saliva (n = 1) samples negative by molecular method, from which pneumococci have been cultured. Crosses represent NPS (n = 227), and saliva (n = 294) samples negative by any method used. The *p*-values reported in panel (**b**) were calculated with McNemar’s test.

Altogether, 158 (40.1%) of 394 children were identified as carriers of pneumococcus by any method used in the study. Ninety-seven children (25.4% of 394) were identified as carriers of *S. pneumoniae* using the conventional culture method (Fig. 1b). Among these 97 children, 94 were positive in NPS and six in saliva. When cultured, NPS samples were more likely to be pneumococcal-positive than saliva samples (McNemar’s, *p* < 0.0001). All 394 saliva samples yielded a colony growth on GENT-agar and all these plates were harvested whereas 68 (17.3%) of 394 NPS cultures were negative for any bacterial growth. In the analysis of results, we treated these 68 NPS samples as negative for *S. pneumoniae* also by molecular method, as previously described^26,27,29,42^. When comparing accuracy of NPS cultures with cultures of saliva and using combined results of a viable *S. pneumoniae* recovery from either NPS or saliva as the reference, the 99.2% (95% CI 97.8–99.8) accuracy of testing of NPS’s was higher than 76.9% (95% CI 71.6–80.3) accuracy of saliva testing (Table 2).

**Table 2:** The accuracy of *Streptococcus pneumoniae* detection in paired saliva samples and nasopharyngeal swabs from n = 394 children.

Method	Sensitivity % (95% CI)	Specificity % (95% CI)	PPV % (95% CI)	NPV % (95% CI)	Accuracy % (95% CI)
Isolation of viable *S. pneumoniae* from culture of NPS	96.9 (91.23–99.36)	100.00 (98.77–100.00)	100.00	99.00 (97.01–99.67)	99.24 (97.79–99.84)
Isolation of viable *S. pneumoniae* from culture of saliva	6.19 (2.30–12.98)	100.00 (98.77–100.00)	100.00	76.55 (75.62–77.45)	76.90 (72.42–80.90)
qPCR on culture-enriched NPS	95.88 (89.66–98.87)	90.57 (86.66–93.64)	76.86 (69.96–82.57)	98.53 (96.26–99.43)	91.88 (88.73–94.38)
qPCR on culture-enriched saliva	53.61 (43.19–63.80)	86.20 (81.74–89.91)	55.91 (47.46–64.03)	85.05 (82.05–87.62)	78.17 (73.76–82.15)

Samples were tested using culture method and molecular methods applied to DNA extracted from culture-enriched (CE) samples and applying thresholds of < 40 C_*T*_ in *piaB*- and *lytA*-specific qPCRs for a sample positivity. Measures of diagnostic accuracy were calculated by comparing the number of samples positive by a method with the overall number of n = 97 children identified as carriers of *S. pneumoniae* based on isolation of viable pneumococcus either from saliva or nasopharyngeal sample.

*NPS* nasopharyngeal swab, *PPV* positive predictive value, *NPV* negative predictive value, *95% CI* 95% confidence interval.

Samples from 155 children (39.1% of 394) were identified as positive for pneumococcus with qPCRs (Fig. 1b). qPCR positives included all samples from which S*. pneumoniae* was cultured except for three NPS samples positive by culture for non-typeable (NT) pneumococci (Fig. 1a), and a single saliva sample from which serotype 24F isolate was cultured (Fig. 1c). Similar to conventional culture, with molecular methods the number of positive results was higher for NPS’s compared to saliva (121 or 30.7% vs. 93 or 23.6%; McNemar’s, *p* < 0.01) (Fig. 1b). However, unlike conventional culture, testing saliva on the top of NPS significantly increased the number of *S. pneumoniae* carriers detected from 121 to 155 (*p* < 0.05). Also, the number of carriers detected by testing the DNA extracted from culture-enriched saliva samples was no different to the number detected by the gold standard of NPS culture (94 or 23.9% vs. 93 or 23.6% of 394; McNemar’s, *p* = 1.0). Of note, 12 of 68 children with negative NPS cultures were positive for *S pneumoniae* in their saliva using qPCR. For NPS’s, the 91.9% (95% CI 88.7–94.4) accuracy of carriage detection with qPCR was lower compared to 99.2% (95% CI 97.8–99.8) accuracy of culture (Table 2). This was primarily due to the molecular method reduced PPV (76.9%, 95% CI 70.0–82.6 vs. 1.000, for qPCR and culture, respectively) and specificity (90.6%, 95% CI 86.7–93.6 vs. 1.000, 95% CI 98.8–100.0, for qPCR and culture, respectively). Despite qPCR having more than an eight-fold increase in the sensitivity of detection; from 6.2% for culture (95% CI 2.3–13.0) to 53.6% for qPCR (95% CI 43.2–63.8), the accuracy of carriage detection with qPCR was only marginally higher than for saliva culture, with 78.2% (95% CI 73.8–82.2) and 76.9% (95% CI 72.4–80.1), respectively. We attributed the poor accuracy of saliva compared to NPS testing to a high density of non-pneumococcal species on saliva culture plates, either masking the detection of pneumococcal colonies or making pneumococci non-viable, as it has been described by Boelsen et al.^32^ for the oropharyngeal samples from adults.

### Risk factors for *S. pneumoniae* carriage

In univariate analysis, the highest risk ratio (RR) of pneumococcal carriage was associated with DCC attendance (RR 2.13, 95% CI 1.43–3.16, *p* < 0.0001, and RR 2.01, 95% CI 1.53–2.64, *p* < 0.0001, when detected by the culture of NPS’s and by any method, respectively, Table 1). Being a sibling was associated with a higher risk of carriage in the study (RR 1.50, 95% CI 1.01–2.23, *p* < 0.05), but only when *S. pneumoniae* was detected by the culture of NPS and was driven primarily by an effect observed in 12–23 month-olds (RR 2.40, 95% CI 1.28–4.51, *p* < 0.001) and not in other age groups (24–35 months old, *p* = 0.70; 36–47 months old, *p* = 0.17; 48–59 months old, *p* = 0.66). When carriage was detected by any method, presence of siblings was associated with elevated risk of carriage for children staying home (RR 2.72, 95% CI 1.41–5.26, *p* < 0.01), yet it has no impact on RR among DCC-attendees (RR 0.98, 95% CI 0.75–1.27, *p* = 0.88). RR was also elevated during the autumn and winter months. In univariate analysis, RR was higher in children from households without a smoker (*p* < 0.05), but only when carriage was detected by the culture of NPS’s. However, with a smoker-associated RR of 1.14 (95% CI 0.74–1.76, *p* = 0.61) for children with no siblings, and RR of 1.33 (95% CI 0.90–1.97, *p* = 0.18) for DCC-attendees among these children, an elevated risk of carriage in households without a smoker could represent confounding bias of child’s social interaction. There was no effect of age or sex on prevalence of carriage detected by a particular method or overall (Table 1).

### Serotype carriage

Serotypes of isolates cultured from children and detected with qPCR in culture-enriched samples in the study are all listed in Table 3. Altogether, 99 isolates were obtained from 97 children, as *S. pneumoniae* isolates of two different serotypes were cultured from NPS’s collected from two individuals. Ninety-three of these 99 isolates represented 26 different serotypes and the remaining six were classified as non-typeable pneumococci. The most common serotype among *S. pneumoniae* isolates were 23A and 6B cultured from 10 children each, followed by 15BC, 10A and 11A isolated from seven children each, and serotypes 23B and 35F isolated from six children each. Isolates of these seven serotypes constituted 53.5% of 99 isolates cultured in the study. The coverage of PCV10 and PCV13 was 23.2% (23 of 99) and 26.3% (26 of 99), respectively.

**Table 3:** Serotypes of strains cultured in the study or detected using serotype-specific and serogroup-specific qPCRs.

Serotype	NPS			saliva			NPS or saliva		
	Culture	qPCR	Overall	Culture	qPCR	Overall	Culture	qPCR	Overall
1^PCV10^	0	1	1	0	0	0	0	1	1
3^PCV13^	2	2	2	0	5	5	2	7	7
4^PCV10^	0	NR^#^	0	0	NR^#^	0	0	NR^#^	0
5^PCV10^	0	NR^#^	0	0	NR^#^	0	0	NR^#^	0
6 (A^PCV13^/B^PCV10^/C/D)	14 (1/10/3/0)	15	16	0	15	15	14 (1/10/3/0)	22	23
7 (A/F^PCV10^)	0	0	0	0	0	0	0	0	0
8	0	0	0	0	0	0	0	0	0
9 (A/L/N/V^PCV10^)	1 (0/0/1/0)	9	9	0	10	10	1 (0/0/1/0)	17	17
10 (A/B)	7 (7/0)	6	7	0	2	2	7 (7/0)	7	8
11 (A/D)	7 (7/0)	10	10	0	8	8	7 (7/0)	14	14
12 (A/B/F)	0	1	1	0	4	4	0	4	4
14^PCV10^	3	4	4	0	4	4	3	6	6
15 (A/BC/F)	13 (6/7/0)	22	22	1 (1/0/0)	12	12	13 (6/7/0)	24	24
16F	0	0	0	0	0	0	0	0	0
18 (A/B/C^PCV10^/F)	1 (0/0/1/0)	1	1	0	0	0	1 (0/0/1/0)	1	1
19A^PCV13^	0	2	2	0	0	0	0	2	2
19F^PCV10^	4	4	4	0	0	0	4	4	4
20	0	0	0	0	0	0	0	0	0
21	1	1	1	0	2	2	1	3	3
22 (A/F)	3 (0/3)	8	8	0	3	3	3 (0/3)	10	10
23A	10	11	12	0	10	10	10	17	18
23B	5	6	6	1	4	5	6	8	9
23F^PCV10^	5	4	5	0	4	4	5	6	6
24F^&^	2	–	2	2	–	2	2	–	2
28F^&^	2	–	2	0	–	0	2	–	2
31^&^	1	–	1	1	–	1	2	–	2
33 (A/F)	0	1	1	0	3	3	0	3	3
34	0	0	0	0	0	0	0	0	0
35A^&^	1	–	1	0	–	0	1	–	1
35B	1	2	2	0	6	6	1	7	7
35F^&^	6	–	6	0	–	0	6	–	6
37^&^	1	–	1	0	–	0	1	–	1
38	1	3	3	0	3	3	1	5	5
NT^@^	5	–	5	1	–	1	6	–	6
Total	96	112	135	6	95	100	99^**a**^	168^**b**^	192^**c**^

^#^An assay of insufficient specificity thus considered to be not reliable (NR), ^&^serotypes not targeted by qPCR, thus detected by culture alone, ^@^non-typeable *S. pneumoniae* strains, ^**a**^number of *S. pneumoniae* strains cultured from 97 carriers, ^**b**^number of serotypes detected in 155 individuals identified as carriers of *S. pneumoniae* by molecular method (qPCR), ^**c**^number of serotypes detected in 158 individuals identified as carriers of *S. pneumoniae* by any method used in the study, ^PCV10^serotype targeted by PCV10 and PCV13, ^PCV13^serotype targeted only by PCV13.

The qPCR assays applied to detect serotype-specific sequences in DNA extracted from culture-enriched samples did cover serotypes of 79 or 84.9% out of 93 encapsulated *S. pneumoniae* isolates cultured in this study. Among remaining 14 isolates were *S. pneumoniae* of serotype 35F (n = 6), 24F, 28F, 31 (n = 2 each), 35A and 37 (single isolate each) (Table 3). Application of qPCR lead to the detection of 168 serotype-carriage events in 124 (78.5%) of 158 of children classified as carriers by any method used. Of these 168 serotype-positive results detected with qPCR, 112 events were detected in 93 NPS’s and 95 events were detected in 68 saliva samples, representing 75.0% of all 124 NPS samples and 72.3% of all 94 saliva samples positive by any method for *S. pneumoniae*. The remaining 57 samples (26.1%) with no signal for any serotype were 31 NPS’s and 26 saliva samples collected from 34 carriers.

**Figure 2:**
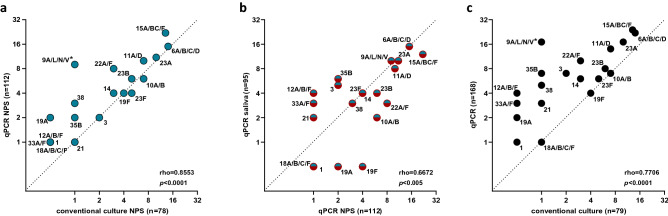
Scatter plot of results of pneumococcal serotypes detected by conventional culture and molecular method (qPCR). Panels (**a**) and (**c**) depict agreement between number of cultured (X-axis) and number of samples positive in qPCR among serotypes or serogroups targeted by qPCR assays. Panel (**a**) depicts results exclusively for NPS samples. Panel (**c**) depicts results for all strains cultured from NPS or saliva versus detected in NPS or saliva in qPCR assays. Panel (**b**) shows agreement between NPS (X-axis) and saliva (Y-axis) for serotypes detected exclusively with qPCR assays. Serotypes not detected using a given approach have been assigned a value of 0.5. The rho, and *p* values have been calculated with Spearman’s test. Asterisks depict significant differences in frequency of positive results between approaches compared *p < 0.05.

Among 158 children classified as carriers of *S. pneumoniae* by any method, the most common serotype/serogroup detected with qPCRs were: serogroup 15 (n = 24 or 15.2% of 158 children), serogroup 6 (n = 22 or 13.9%), serogroup 9 and serotype 23A (n = 17 or 10.8%, each). Since the number of serotype carriage events detected with qPCR was higher compared to the number of isolates of corresponding serotypes cultured form children (n = 112 vs. n = 78 for NPS, n = 95 vs. n = 2 for saliva, and n = 168 vs. n = 79 for overall), we tested if any individual serotype or serogroup was overrepresented in qPCR results. The only significant difference between the rate of samples positive in qPCR compared to the rate of corresponding serotype/serogroup isolates cultured was observed for serogroup 9 (Fig. 2a,c). With that in mind, there was an overall agreement between the rates of samples positive for serotype/serogroup via molecular method versus culture (Fig. 2). For NPS’s, the number of samples positive for serotype or serogroup by culture were concordant with the number of samples positive for the same serotypes by qPCR (Spearman’s rho = 0.855, *p* < 0.0001) (Fig. 2a). There was also agreement between the number of serotype carriers detected with qPCR in NPS’s and in saliva (Spearman’s rho = 0.667, *p* < 0.01) (Fig. 2b), as well as between the numbers of serotype-carriers detected with qPCR in NPS’s or in saliva versus overall cultured in the study (Spearman’s rho = 0.771, *p* < 0.0001) (Fig. 2c).

Although the absolute number of VT isolates cultured from children attending DCC was significantly higher compared with children staying home only for PCV13-VT (n = 20 isolates cultured from 207 DCC attendees vs. n = 6 isolates cultured from 187 children staying home, *p* < 0.05), there were no differences for PCV10-VT (n = 17 isolates cultured from 207 DCC attendees vs. n = 6 isolates cultured from 187 children staying home, *p* = 0.051) and in fractions of VT isolates among all n = 71 cultured from DCC attendees compared with fractions among all n = 28 cultured from children staying home (17/71 or 24.0% vs. 6/28 or 21.4% for PCV10-VTs, *p* = 1; and 20/71 or 28.2% vs. 6/28 or 21.4% for PCV13-VTs, *p* = 0.62). Although the DCC attendees were more likely to be carriers, the serotype distribution was not different between children attending DCC and staying home. None of the other demographic or environmental factors were associated with differences in serotype carriage.

### Co-carriage of multiple serotypes

The presence of two or more serotypes was detected in 40 (25.3%) of 158 children identified as carriers by either culture or using *piaB* and *lytA* qPCRs (Table 4). Co-carriage of two or more serotypes/serogroups was detected in 17.7% (22 of 124) NPS’s and 25.5% (20 of 94) saliva samples classified as positive for *S. pneumoniae* by any method. There was no significant difference in the prevalence of co-carriage in NPS’s and saliva samples (*p* = 0.60). Also, there were no statistically significant differences in co-carriage according to age (12–23 months old, 17 of 62; 24–35 months old, 6 of 36; 36–47 months old 12 of 30; 48–59 months old, 5 of 30; *p* > 0.05 between any two age groups), DCC attendance (‘yes’ 32 of 109 vs. ‘no’ 8 of 49, *p* = 0.11), having siblings (‘yes’ 23 of 105 vs. ‘no’ 17 of 53, *p* = 0.18) nor the presence of a smoker in the child’s household (‘yes’ 15 of 48 vs. ‘no’ 25 of 110, *p* = 0.32).

**Table 4:** Number of serotypes/serogroups detected with any method.

Number of detected serotypes	NPS	Saliva	Overall
0	270	300	236
1^#^	102	74	118
2	19	13	29
3	3	5	8
4	0	2	3

^#^Includes samples pneumococcus-positive for *S. pneumoniae* in qPCR with no signal for any serotype.

### Temporal changes in serotype carriage

To assess the impact of the implementation of PCV into the NIP in January 2017 on the VT serotypes carriage in unvaccinated children, we tested for temporal changes in the fraction of PCV10-VTs and PCV13-VTs among cultured pneumococci. We assumed that herd effects of PCV implementation will lead to a decrease of VT carriage after the vaccine roll-out. However, there were no significant differences in the rate of PCV10-VTs and PCV13-VTs before (4/30 or 13%, for both PCVs) and after January 2017 (19/69 or 28% and 22/69, or 32% respectively). Instead, there was a trend towards an increase in the VTs fraction over time (*p* = 0.19 and *p* = 0.08 for PCV10-VTs and PCV13-VTs, respectively). Also, there were no differences in the fractions of PCV10-VTs and PCV13-VTs up to (8/50 or 16% and 9/50 or 18%, respectively) and after (15/50 or 30% and 17/50 or 34%, respectively) the 50th of 99 isolates had been cultured in the study in October 2017. The trend towards an increase in VTs fraction over time was still present (*p* = 0.15 and *p* = 0.11 for PCV10-VTs and PCV13-VTs, respectively).

## Discussion

There is little data published on pneumococcal carriage in Poland. Over the past two decades reports of only two Polish studies investigating the carriage of *S. pneumoniae* have been published. The first study was conducted between November 2000 and May 2001 in the capital city of Warsaw^15,16^. The second study took place between November 2002 and June 2003 in Lublin^17,18^. Both these studies have been conducted before the first of the PCVs, the heptavalent vaccine, became available commercially in the country. In light of obligatory pneumococcal vaccination for children born after the 31st of December 2016, data on pneumococcal carriage is needed. Here, we investigated *S. pneumoniae* carriage in unvaccinated children under five years of age in the country with PCV10 and PCV13 commercially available for over five years and at the time of PCV10 implementation into the infants’ immunization program. Our first goal was to establish a baseline for future studies investigating the impact of PCV10 introduction into NIP on *S. pneumoniae* carriage. The second goal was to assess the suitability of molecular methods and of testing oral fluids for pneumococcal carriage detection by comparing saliva testing with the gold standard method of conventional culture of NPS.

Conventional culture was the only method used to detect *S. pneumoniae* in previous studies conducted in Poland between 2000 and 2003^15–18^. The carriage rate of 25.4% we detected with that method in our cohort was lower compared with 45.7–54.6% reported by Sulikowska et al*.*^15,16^ and 33.1–44.4% by Korona-Glowniak et al.^17,18^. However, the differences in cohorts’ demographic composition, methods of sampling, and seasons of sample collection, make interpretation of differences in *S. pneumoniae* carriage difficult.

It is documented that DCC attendance increases pneumococcal transmission^43–45^. In all three Polish studies, which includes this study, the risk of pneumococcal carriage was significantly higher in children attending DCCs compared to staying at home. Hence, the higher proportion of children staying home in our study (47.5%) compared with the cohort investigated by Sulikowska et al*.*^15,16^ (25.7%) and by Korona-Glowniak et al.^17,18^ (22.3%), per se contributed to lower rate of *S. pneumoniae* carriage. Interestingly, whereas carriage rate of 31.9% detected by us with conventional culture of NPS’s in children attending DCCs was significantly different from 54.2–56.5% by Sulikowska et al*.* (*p* < 0.01), there was no significant difference between the rate of 15.0% we detected in home-setting children compared with 19.3–25.9% reported by Sulikowska et al.^15,16^ (*p* > 0.1). Also, children were enrolled into our study all year long whereas in the previous two, exclusively during autumn and winter, the seasons when carriage rates are higher^45^. It seems very likely that the continuous enrolment contributed further to lower carriage rates in our study. Finally, higher *S. pneumoniae* carriage reported by Korona-Glowniak et al.^17,18^ can be attributed to oropharyngeal sample (OPS) being cultured on the top of NPS in their study. When excluding results for OPS, there was no difference in point-prevalence of 25.4% detected with NPS’s culture by us and 24.4% detected by Korona-Glowniak et al.^17,18^ (n = 94 of 394 vs. n = 228 of 933, Chi-square, *p* = 0.88).

There was, however, a reduction in proportions of PCV10-VTs (23.2% vs. 73.4%) and PCV13-VTs (26.3% vs. 80.4%) among the isolates cultured in our study, when compared to the 2002–2003 study conducted by Korona-Glowniak et al.^17,18^ prior to PCVs entering the Polish market. This reduction in VTs carriage in unvaccinated children is likely to represent a herd effect of commercial vaccination with PCVs prior to and independent from PCV10 implementation into the NIP. Interestingly, instead of a decline of PCV10-VTs carriage over the study period there was, albeit not statistically significant, an increase in a fraction of VTs among all isolates cultured. Absence of decline in VT carriage implies that our study can be considered to represent the baseline for future assessment of effects of PCVs introduction into NIP on carriage.

One noticeable result was the low prevalence of serotype 19A in carriage. Serotype 19A has been reported to emerge at various sites in replacement after PCV7 implementation^8,39^ and to persist in carriage in populations with infants vaccinated with PCV10^42,46^. Here, carriage of 19A was detected in only two children and exclusively with the molecular method. With estimates of 25–30% of infants being vaccinated in Poland with PCV13 outside the NIP, the low presence of this PCV13-VT could reflect PCV13 herd effects. In our study serotype 19A ranked 19th in frequency in carriage, whereas between 2016 and 2020 (time of present study) 19A was, after serotype 14, the second most common in IPD in Polish children aged 12–59 months^7^. It indicates a high IPD cases to carriers’ rate, thus high invasiveness of serotype 19A strains circulating in Poland.

Importantly, low numbers of VTs cultured from unvaccinated children make future assessment of direct effects of PCV implementation into NIP on VTs carriage difficult. The solution could be increasing the power of the next study by sampling higher numbers of subjects and/or detecting carriage of serotypes with a substantially more sensitive approach. The latter can be addressed by testing multiple samples per child^17,18^ and/or employing molecular detection methods.

In our study, application of molecular method and testing saliva on the top of NPS’s increased the number of carriers detected by a factor of 1.7, from ninety-four identified by gold standard of conventional culture of NPS’s to an overall number of 158. In both materials, NPS and saliva, the application of molecular methods significantly increased the sensitivity of *S. pneumoniae* detection. This is in line with results reported by Wyllie et al*.* in studies applying a similar protocol to test NPS’s from children and to test NPS’s and saliva samples from adults, conducted between 2014 and 2016 in the Netherlands^21,42^.

NPS is the specimen recommended by WHO in pneumococcal carriage detection in children^20^ and it has been reported that NPS is a more valuable material than OPS^47^. Also in our study, the culture of NPS’s was far superior to culture of saliva and 39.6% of carriers (61 of 154) identified by qPCR were detected by NPS’s only. However, with 21.5% (34 of 158) of carriers detected exclusively in saliva, testing oral fluids substantially increased the number of carriers detected. In line with this finding, Korona-Glowniak et al.^17,18^ reported that testing OPS along NPS’s significantly increased the number of carriers detected, and that there was no difference between the number of carriers detected by culturing NPS compared with OPS^17,18^. Therefore, the optimal carriage detection might be achieved by testing from each individual multiple specimens, e.g. NPS, OPS and saliva, or a combination of any two of these.

Molecular methods appeared to be superior to conventional culture in detecting co-carriage of multiple serotypes in this study (2.1% in culture vs. 26.6% in qPCR). Wyllie et al*.*^42^ and Kandasamy et al*.*^48^ obtained similar levels of multiple serotype carriage using molecular methods. The higher sensitivity of any minority serotype detection in multi-serotype carriage allowed for a more detailed analysis of the occurrence of serotypes. Since available qPCR assays did not cover all serotypes, and not always distinguished serotypes within a serogroup, the number of multi-serotype carriers still might be understated.

Among our study limitations was a lack of molecular assays that would detect carriage of every circulating serotype. For example, isolates of serotypes 24F, 28F, 35A, 35F, and 38 have been cultured from children, yet none of these serotypes were targeted with qPCR. Another limitation was low resolution of certain qPCRs not discriminating between serotypes within a serogroup, with 10 out of 27 qPCR assays targeting more than one serotype. A limitation was also the low sensitivity of conventional culture. It concerns both sample types, but due to very rich bacterial growth, including many non-pneumococcal α-hemolytic colonies, it was particularly difficult to culture *S. pneumoniae* from saliva. With large numbers of serotype-carriage events detected exclusively with qPCR, and in the light of reports on non-pneumococcal streptococci expressing the pneumococcal capsular polysaccharides^7,49^, we paid particular attention to the specificity of assays. We addressed it by testing for serotype samples negative for *S. pneumoniae* and excluding the results of assays that generated a positive result (serotype 4 and serotype 5 specific qPCRs)*.* Nevertheless, we can’t exclude that some of the results represent carriage of confounded non-pneumococcal bacteria detected with qPCRs. For example, when applied to oropharyngeal and saliva samples from adults, a diminished specificity of serogroup 9-specific assay has been reported^27,37^ and serogroup 9 was the clear outlier when culture data was compared with qPCR results in our study (Table 3, Fig. 2a,c). However, since we did not observe positivity in this assay among samples negative for *S. pneumoniae*, nor was there a difference between the number of NPS’s and saliva samples positive for this serogroup by qPCR, and we are not aware of any reports on the assay’s poor specificity in NPS’s from children, we consider results for serogroup 9 as reliable. Finally, we acknowledge that the exclusion from analysis of performance the serotypes cultured yet not targeted by qPCRs and serotypes 4 and 5 not cultured yet targeted by assays of poor specificity may result in bias that favors molecular detection.

In summary, pneumococcal carriage rate detected in Polish children was lower compared with studies conducted prior to the introduction of commercial PCVs in the country, yet we attribute it to differences in set-ups of studies rather than the effect of PCVs. On the other hand, the decline in prevalence of PCV10-VTs and PCV13-VTs carriage compared with the pre-PCV period suggests strong herd effects of commercial vaccination independent of NIP in Poland. According to the results obtained in our study, NPS was a more valuable material in carriage detection in children and qPCR was the more sensitive method in pneumococcal and pneumococcal serotype carriage detection. Also, information about carriage rate and serotype distribution among unvaccinated Polish children obtained during this study can be used as a baseline in future carriage projects. The knowledge concerning the methods used in pneumococcal carriage detection gained during our study can be used for further research.
